# Persistent misunderstandings about evidence-based (sorry: informed!) policy-making

**DOI:** 10.1186/s13690-016-0142-z

**Published:** 2016-07-20

**Authors:** Pierre-Olivier Bédard, Mathieu Ouimet

**Affiliations:** Department of Economics, Université Laval, 1025, Avenue des Sciences-Humaines, Québec, G1V 0A6 Canada; Department of Political Science, Université Laval, 1030, Avenue des Sciences-Humaines, Québec, G1V 0A6 Canada

**Keywords:** Evidence-based policy-making, Research methods, Critical rationalism

## Abstract

**Background:**

The field of research on knowledge mobilization and evidence-informed policy-making has seen enduring debates related to various fundamental assumptions such as the definition of ‘evidence’, the relative validity of various research methods, the actual role of evidence to inform policy-making, etc. In many cases, these discussions serve a useful purpose, but they also stem from serious disagreement on methodological and epistemological issues.

**Discussion:**

This essay reviews the rationale for evidence-informed policy-making by examining some of the common claims made about the aims and practices of this perspective on public policy. Supplementing the existing justifications for evidence-based policy making, we argue in favor of a greater inclusion of research evidence in the policy process but in a structured fashion, based on methodological considerations. In this respect, we present an overview of the intricate relation between policy questions and appropriate research designs.

**Summary:**

By closely examining the relation between research questions and research designs, we claim that the usual points of disagreement are mitigated. For instance, when focusing on the variety of research designs that can answer a range of policy questions, the common critical claim about ‘RCT-based policy-making’ seems to lose some, if not all of its grip.

## Background

Under the impulse of evidence-based medicine, evidence-based policy-making now appears to have a life on its own. Even more so, evidence-based policy-making, as a slogan, is now conveniently tossed around by academics and policy actors, sometimes purporting ill-defined meanings. And it is not to say that there haven’t been enough writings about it. To this day, a considerable amount of papers, books and reports have been published – from either a theoretical, empirical and/or normative stance: countless articles devoted to defining what evidence-based policy-making is and is not, what levels of success (or lack thereof) have been reached in implementing evidence-based principles and practices in policy-making, what barriers need to be overcome to fruitfully implement these principles (see, for instance, the latest systematic review [1]), etc. To be sure, a lot more needs to be done – the field(s) of research underlying evidence-based policy-making is(are), after all, still in its(their) infancy.

In parallel, one still frequently comes across the usual reluctant, skeptical and *all-things-evidence* pessimist – something we erroneously took for granted to be a bygone figure. Then again, a lot has been written both in defense and in reaction to evidence-based policy-making and its normative and practical implications. Indeed, some substantial misunderstandings persist, likely fueled by methodological disputes and epistemologically conflicting views about the role of science in society, in general, and in the policy arena more specifically.

Our objective here is not to write a definitive defense of evidence-based policy-making but to try and dismiss some of the misunderstandings about it. Our motivation stems from the fact that these conflicting views are in our view the outcome of two intertwined factors: 1) an unsatisfactory portrayal by advocates of evidence-based policy-making; and 2) a misleading depiction by skeptics.

Our argument revolves around these lines: advocates of evidence-based policy-making have yet to present a nuanced, epistemologically realist and methodologically substantive perspective; and skeptics have yet to embrace the idea for fear of improbable (and almost unreasonable) implications. We won’t make here the customary distinctions between evidence-based and evidence-informed. But, as shall be clear from this article, what we can expect from evidence – even at best – is a supporting role in the policy-making process. More specifically, we present a detailed view on how relevant research can be sorted out beforehand – and beyond surveying hierarchies of evidence – by examining the adequacy between types of questions in need of evidence and the research designs most likely to provide relevant and valid answers.

## Discussion

### Evidence-based everything?

From its gradual emergence as a potentially viable and fruitful idea, evidence-based policy-making has prompted some reservations. From technocratic suspicions [2], to evocations of restrictive ‘RCT-based policy-making’[3], the overall unreliability of scientific results and to the possibility of doing more harm than good when it comes to health policy interventions [4–6], the idea of evidence-based policy-making seems to have been hashed out from just about every angle.

As in any area of practical rationality, it is rather unlikely that evidence can be the only decisional factor. Under practical constraints and limited rationality, realistic expectations can only go so far as to make valid and relevant evidence one of the factors weighing in a decision process and, at best, a weighty justification for a specific course of action. On the other hand, and this is possibly where evidence-based policy-making has a strong case, policy processes can be more or less informed by evidence. Alternatively, they can be completely uninformed by evidence, thus giving weight to other sources of justification (opinions, interests, gut feelings, etc.). Evidence-based policy-making, rather than trying to turn everything into an occasion to delve into scientific literatures, has a clear answer to these alternatives scenarios: one should try and find the most reliable, most objective, most relevant evidence available and make the most out of it within practical constraints.

Inasmuch as evidence-based policy-making requires the interplay of knowledge generation (by scientists and/or academics) and public decision-making processes, it is likely to be a complex enterprise. One obvious reason is that both processes are complex enough on their own and commonly take place independently of one another. As Black [7] rightfully warns us:

Researchers need a better understanding of the policy process, funding bodies must change their conception of how research influences policy, and policy makers should become more involved in the conceptualisation and conduct of research. Until then, researchers should be cautious about uncritically accepting the notion of evidence based policy

This appears entirely reasonable in that it does not discredit the principles themselves, but rather sheds light on their difficult implementation and provides realist expectations about the success rate of evidence-based policy-making practices (i.e. they do not always work and they are not ready-made solutions to every policy problem).

So, what does it really imply? Multiple things, mainly along two lines: 1) behavioral change from policy actors and the adaptation of the policy-making process, and 2) behavioral change from researchers in their way of communicating research evidence. On the one hand, proper infrastructures and resources need to be in place to support evidence-based principles [8, 9] and the decision-making process need some adjustments to allow a greater input from research evidence. On the other hand, further attention should be directed at the level of proof. In this respect, hierarchies of evidence typically display a structured view of the internal validity of research designs when pursuing causal inference. However, we believe that the relation between evidence and the policy process needs to be even more elaborated and structured so as to reflect the variety of research questions policy-makers are interested in.

No single design can answer everything, not even RCT’s [10]. Turning evidence gathered in one context into policy advice in *another context* is not straightforward, not even with well executed RCT’s [11]. The question of transferability of results is an important and difficult one, but tools exist to guide this process [12, 13]. In sum, even in the specific context of causal inference, RCT’s are not the only research design that can claim to validly inform decision, as they can themselves be controversial, contradictory or irreproducible. As Chalmers [5] puts it: “any engagement with empirical evidence needs to be serious, not polemical”. We believe the seriousness of the engagement should reflect a thorough discussion (well beyond the scope of this article) regarding the adequacy of a given research design to the questions on is interested in.

### Problem-based policy processes and relevant research designs

In the policy arena, policy-makers typically pursue a wide variety of policy objectives and are in need of multiple sources of information and evidence. In science, researchers are attentive to questions of efficacy and causal inference, but also other types of questions. Analogously, policy-makers are interested in answering different kinds of questions, where matters of causal inference are just one of them. If one is interested in evaluating the efficacy of a given potential intervention, no doubt that surveying RCT’s (of syntheses of them) are your first best bet. But if you are interested in something else, or should you find yourself facing a lack of such evidence, you have to turn to different research designs. This is where evidence-based policy-making advocates generally leave potential research users without much clue, perhaps giving the impression that RCT’s are the overall, wide-ranging gold standard. As argued by Davies & Powell [14] “the knowledge requirements for effective social policy and effective service organisation go far wider than just ‘what works”’. We couldn’t agree more, but a potential guide to the principled inclusion of other research designs in the policy process is still lacking.

As is generally the case in science, the research process usually starts by defining a problem and accordingly formulating specific questions. The next logical step is to identify and perhaps devise the proper research design to answer your question. We argue here that policy-makers should probably proceed in a similar fashion when looking for evidential support.

Policy-makers can look into a variety of types of questions in need of answers. The can be broadly categorized as questions related to 1) specific interventions, 2) non-interventional determinants or, 3) specific outcomes. For each of these lines of questioning, a wide array of research question can be elaborated. For example, if one is interested in a specific intervention, say a policy to increase access to health services, one can be interested in the effectiveness of the intervention or in the views of different stakeholders. In cases of questions pertaining to effectiveness, the ideal research design to provide such answer is undoubtedly an experimental design (such as an RCT) or if unavailable a quasi-experimental one. In the case of stakeholder’s views on the said intervention, experimental and quasi-experimental designs are not needed, but rather designs such as cross-sectional opinion surveys, qualitative studies using interviews or mixed-methods are appropriate. In other words, each type of question can be linked to an appropriate research design to be prioritized. Doing so offers a complement to traditional hierarchies of evidence and provides more detail and guidance for end user’s such as policy-makers.

Table 1 presents a non-exhaustive list of types of questions and of research designs that would ideally provide each type of question with the most reliable and valid answers. In a recent unpublished rapid review from one of the co-authors (MO) and colleagues conducted on behalf of a division of a Canadian ministry whose analysts wanted to learn about the phenomenon of skills mismatch, the list of 11 questions was presented to the client at the start of the project to assess their needs. The client selected seven questions that were deemed relevant to him. This example perfectly illustrates that policy-relevant questions extend way beyond matters about effectiveness and that all kinds of research studies might at one point be needed.

**Table 1:** A non-exhaustive list of types of questions and research designs

Research questions	Research designs to be prioritized
**A. Questions about interventions**	
Is the intervention effective and/or harmful?	Intervention under the control of the researchers/evaluators
	(usually for short-term assessment)
	∘ Randomized controlled experiments
	∘ Prospective non-randomized controlled experiments
	(no pure randomization procedure)
	∘ Controlled-before-and-after study
	Intervention NOT under the control of the researchers/evaluators
	and/or long term effectiveness data are needed (usually for long term assessment)
	∘ Interrupted time series
	∘ Prospective cohort studies
	∘ Case-control studies
	∘ Cross-sectional studies
Why is the intervention ineffective or less effective than expected?	∘ In depth mixed-method case studies
	∘ In depth qualitative case studies
	∘ Cross-sectional studies
What are the stakeholders’ opinions and views about the intervention?	When targeted stakeholders are numerous and from different jurisdictions, organizations or organizational units AND high external validity is targeted
	∘ Cross-sectional opinion surveys
	When targeted stakeholders are few and located in few settings
	∘ In-depth mixed-methods studies including an opinion survey and
	qualitative interviews
	∘ In-depth qualitative studies with interviews only
Is the intervention cost-effective?	∘ Formal economic evaluations
What are the characteristics of the intervention?	∘ Any type of studies that include an objective and a factual description of the intervention.
Is the intervention transferable to another context?	∘ Specific components of the study (e.g. population, environment, etc.) and the comparability of contexts need to be systematically assessed.
**B. Questions about non-interventional correlates of specified outcome(s)**	
Are correlates associated with the outcome(s)?	∘ Prospective cohort studies
	∘ Retrospective cohort studies
	∘ Case-control studies
	∘ Cross-sectional studies
To what extent are correlates associated with the outcome(s)?	∘ Prospective cohort studies
	∘ Retrospective cohort studies
	∘ Case-control studies
	∘ Cross-sectional studies
Why correlates might be (or might not be) associated with the outcome(s)?	∘ Studies reporting the results of an empirical test of two (or more)
	competing theoretical explanations
	∘ Studies reporting the results of an empirical test of solely one
	theoretical explanation
	∘ Narrative, opinion and textual papers (including formal theorizing
	or not) reporting untested theoretical explanation(s)
**C. Questions about specified outcome(s)**	
What is the prevalence of the outcome?	∘ Cross-sectional studies
What is the incidence of the outcome?	∘ Prospective cohort studies
	∘ Retrospective cohort studies
	∘ Panel studies
What is the economic burden of the outcome?	∘ Any studies reporting validated estimates of the economic burden of
	the outcome for the targeted time frame, population and settings
How important is the outcome for stakeholders?	If targeted stakeholders are numerous and from different jurisdictions, organizations or organizational units AND high external validity is targeted
	∘ Cross-sectional opinion surveys
	If targeted stakeholders are few and located in few settings
	∘ In depth mixed-methods studies including an opinion survey and
	qualitative interviews
	∘ In depth qualitative studies with interviews only

Looking at the table, one first needs to figure out what kind of question needs to be answered and can then look for methodologically grounded suggestions for research designs to look for. For instance, cross-sectional studies should not be your first go to place when interested in causal inference (assuming other types of evidence is available), but cross-sectional surveys might be your best bet to figure out what views stakeholder have of a given intervention. The key here is not to be rigid about which design should be implemented or consulted, but rather to take on board the fact that some designs are more appropriate than others (all things being equal) to answer certain types of question. This principle is quite simple, and to some extent well known by methodologists, but not necessarily cashed-out for policy purposes.

### Evidence-based policy-making and critical rationalism

Here is a list of commonly encountered objections that we mostly agree with but still believe need to stop being put forth as justifications *against* evidence-based policy-making principles, or otherwise need being addressed in a more constructive manner:

- Randomized controlled trials are not the only valid nor relevant research design for policy-making purposes;
- There is a crucial distinction between internal and external validity, such that results in a given context are not always relevant for another context;
- Typical hierarchies of evidence do not say much about how to apply research evidence in the policy process;
- Moreover, one can hardly find a proper guide dealing with how one should actually convert evidence into policy advice;
- Evidence, in itself, even when valid and relevant, is hardly enough to justify a particular course of action. Public policy covers multiple dimensions and some considerations are overly important in a democratic context (i.e. values, feasibility, costs, ethics, etc.) – never to be superseded by evidential matters;
- And, even when trying really hard, you are likely to face a lack of evidence to inform you on a given subject.

Even though that last remark might instill some disappointment in the evidence-based enthusiast, it is still illustrative of the added value of evidence-based practices. It just goes to show that the actual process is perhaps what defines a policy procedure to be evidence-based, rather than its single output. Along with Chalmers [5] we agree that:

A lack of empirical evidence supporting opinions does not mean that all the opinions are wrong or that, for the time being, policy and practice should not be based on people’s best guesses. On matters of public importance, however, it should prompt efforts to obtain relevant empirical evidence through evaluative research, to help adjudicate among conflicting opinions.

## Summary

Going back to the dilemma expressing two possibilities in the policy process, we here reiterate that the general aim of evidence-based policy-making is to go beyond vested interests, conventional wisdom, preconceived notions, opinions, perceptions, etc., and to ensure greater influence of reliable evidence on policy advice. Then again, the idea is to instill a greater dose of rationality into the policy-making process by taking into account relevant research findings – not to uncritically import everything considered as evidence into the policy process. In short, evidence-based policy has nothing to do with scientism: its aim is rather to make the policy-making process less romantic (i.e. in the philosophical sense, purporting an idealized view of reality) and more influenced by reason combined with empirical evidence. The following quote from Gambrill [15] brilliantly summarizes the kind of attitude that advocates of evidence-based policy aim to prevent and minimize:

Censoring lack of evidence for services used, wanting professionals such as physicians and dentists who we consult in our personal lives to base decisions on rigorous criteria when we do not do so for our clients [the population – added by us], hiding methodological limitations, and presenting sloppy reviews of the literature as evidence based all fail to honor ethical obligations described in professional codes of ethics.

Here also, a parallel with science is possible where critical rationalism fuels research practices. Taken from philosopher K.R. Popper [16, 17] this idea of critical rationalism simply suggests that criticism and severity (i.e. considerable caution) towards scientific claims are your best safeguard against false claims and is the proper mindset to secure the advancement of knowledge. To some extent, we see in evidence-based policy-making perhaps the extension of critical rationality principles into another sphere, that is, the policy world. And just as Popper was a fierce opponent of dogmatism and scientism, we advice that these are best left out of the policy-process and, for that matter, of the debate on the merits of evidence-based policy-making.

## Abbreviation

RCT, randomized controlled trial
